# Novel CAD Diagnosis Method Based on Search, PCA, and AdaBoostM1 Techniques

**DOI:** 10.3390/jcm13102868

**Published:** 2024-05-13

**Authors:** Can Eyupoglu, Oktay Karakuş

**Affiliations:** 1 Department of Computer Engineering, Turkish Air Force Academy, National Defence University, Istanbul 34149, Türkiye; caneyupoglu@gmail.com; 2 School of Computer Science and Informatics, Cardiff University, Cardiff CF24 4AG, UK

**Keywords:** AdaBoostM1, cardiovascular diseases, coronary artery disease diagnosis, machine learning, PCA, search techniques

## Abstract

**Background:** Cardiovascular diseases (CVDs) are the primary cause of mortality worldwide, resulting in a growing number of annual fatalities. Coronary artery disease (CAD) is one of the basic types of CVDs, and early diagnosis of CAD is crucial for convenient treatment and decreasing mortality rates. In the literature, several studies use many features for CAD diagnosis. However, due to the large number of features used in these studies, the possibility of early diagnosis is reduced. **Methods:** For this reason, in this study, a new method that uses only five features—age, hypertension, typical chest pain, t-wave inversion, and region with regional wall motion abnormality—and is a combination of eight different search techniques, principal component analysis (PCA), and the AdaBoostM1 algorithm has been proposed for early and accurate CAD diagnosis. **Results:** The proposed method is devised and tested on a benchmark dataset called Z-Alizadeh Sani. The performance of the proposed method is tested with a variety of metrics and compared with basic machine-learning techniques and the existing studies in the literature. The experimental results have shown that the proposed method is efficient and achieves the best classification performance, with an accuracy of 91.8%, ever reported on the Z-Alizadeh Sani dataset with so few features. **Conclusions:** As a result, medical practitioners can utilize the proposed approach for diagnosing CAD early and accurately.

## 1. Introduction

**Figure 1:**
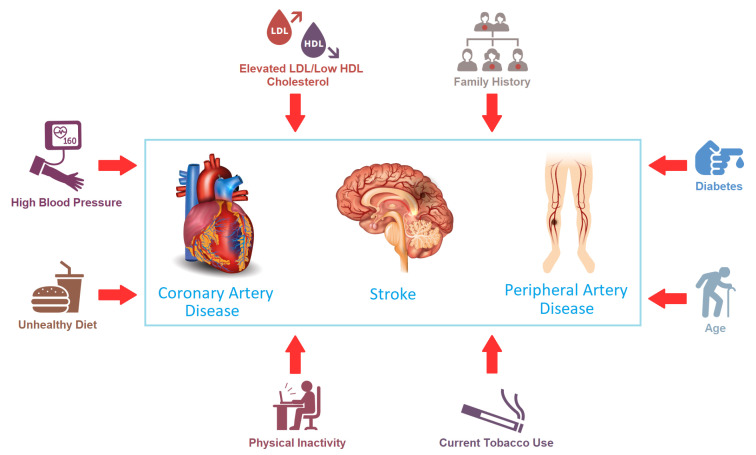
The main types (rectangle at the centre) and risk factors (around the rectangle with arrows) of CVDs [2].

Cardiovascular diseases (CVDs) are a class of disorders that include the blood and heart vessels [1,2]. The main types and risk factors of CVDs are shown in Figure 1. Coronary artery disease (CAD) is an illness that influences the blood vessels providing blood to the heart and occurs when coronary arteries are blocked or narrowed. Additionally, it is associated with ischemic heart disease, coronary heart disease, atherosclerotic heart disease, heart failure, heart attack, sudden coronary death, and angina pectoris medical science. A stroke is disease-causing damage to a particular brain area and occurs when blood vessels are ruptured or blocked. Finally, peripheral artery disease affects the blood vessels that supply blood to the feet and legs [2]. Readers are referred to [2] for the main risk factors of CVDs and characteristics that can be associated with CVDs.

**Figure 2:**
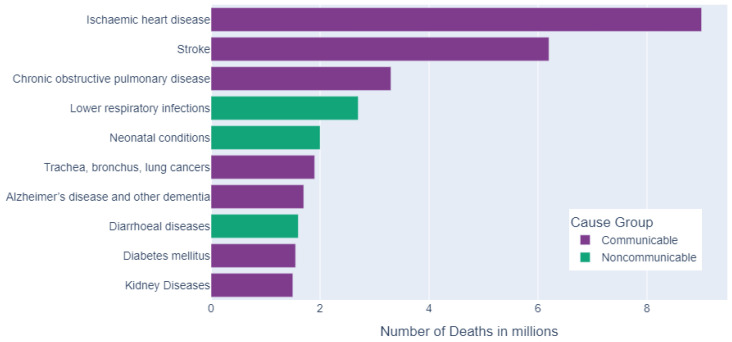
Top ten global causes of mortality in 2019 [4].

CVDs are the main cause of death all over the world. In 2021, the World Health Organization (WHO) [3] reported that nearly 17.9 million people died from CVDs in 2019. This number of mortalities constitutes 32% of the total deaths worldwide, and 85% of these deaths are caused by heart attack and stroke. In addition, the top ten global causes of mortality in 2019 are shown in Figure 2. As shown in the figure, CAD, also known as ischemic heart disease, is the leading reason for deaths [4].

Disease diagnosis is a highly complex process in medical science, and many tests are necessary for accurate diagnosis. In order to help medical doctors with the early detection of disease, machine learning and data mining techniques have been widely utilized recently [5]. Especially in CAD, with early detection, the possibility of treatment is greatly increased and patients’ lives can be saved.

In the literature, numerous methods have been developed to diagnose CAD on the Cleveland heart disease dataset [6] up to the present [7,8,9,10,11,12,13,14,15,16,17]. The prediction and diagnosis success of the existing studies tested on this dataset is quite satisfying. For this reason, in this study, the performance of the proposed approach is evaluated on a newer dataset called the Z-Alizadeh Sani [18], released in 2017. In the work introduced by Alizadehsani et al. [19], the Z-Alizadeh Sani dataset was collected and utilized for the first time for CAD diagnosis. From 2012 to 2016, Alizadehsani et al. employed various machine learning techniques such as sequential minimal optimization (SMO), artificial neural network (ANN), support vector machine (SVM), Naïve Bayes, bagging, C4.5 decision tree, information gain, and a genetic algorithm for CAD detection [19,20,21,22,23,24,25]. In a study using the same dataset, Qin et al. [26] presented a CAD detection method utilizing an ensemble algorithm based on multiple feature selection (EA-MFS) and SVM. Arabasadi et al. [27] proposed a hybrid CAD prediction approach combining a genetic algorithm and multilayer perceptron artificial neural network (MLP-ANN) on a subset of the Z-Alizadeh Sani dataset from which 22 features were selected. In order to diagnose CAD, Babič et al. [28] offered a predictive and descriptive analysis. They used four different classifiers such as decision trees, Naïve Bayes, SVM, and ANN.

In the work of Kılıç and Kaya Keleş [29], the artificial bee colony (ABC) algorithm and SMO technique were utilized for feature selection and classification, respectively. Sixteen features were selected by the ABC algorithm, and SMO was applied to these features. Hu et al. [30] proposed two methods, namely, minimum message length finite inverted Beta-Liouville mixture (MML-IBLMM) and variational finite inverted Beta-Liouville mixture (Var-IBLMM), and then tested the performances of these models on Z-Alizadeh Sani dataset. In the study introduced by Abdar et al. [31], a CAD detection technique called N2Genetic-nuSVM, which is based on a genetic optimizer and nu-support vector classification, was presented. In another work realized by Abdar et al. [32], a nested ensemble nu-support vector classification (NE-nu-SVC) approach was proposed to diagnose CAD accurately. In the feature selection step of the proposed approach, a genetic search method was utilized, and 16 features were selected. In the research of Joloudari et al. [33], the performances of SVM, chi-squared automatic interaction detection (CHAID) decision tree, C5.0 decision tree, and random trees were investigated for CAD diagnosis. The experimental results indicate that the random trees technique is better than the other classifiers. On the other hand, Nasarian et al. [34] presented a hybrid feature selection method named heterogeneous hybrid feature selection (2HFS) that utilizes the synthetic minority over-sampling technique (SMOTE) and adaptive synthetic (ADASYN) for handling the Z-Alizadeh Sani dataset and uses random forests, Gaussian Naïve Bayes, eXtreme Gradient Boosting (XGBoost), and decision tree for CAD classification. In another work proposed by Ashish et al. [35], a CAD detection method based on random forests, SVM, and XGBoost was introduced. In the data-dividing step of the method, the random forests technique was used for training and testing of the Z-Alizadeh Sani dataset. In the classification step, the SVM and XGBoost techniques were utilized together. In a recent study [36], an ensemble feature selection approach and seven classifiers were used, and the best classification accuracy rate was attained with 25 features and the MLP classifier.

The aforementioned studies adopted some combination of feature selection, feature extraction, and classification techniques such as information gain, genetic algorithm, ABC, bagging, decision trees, random trees, Naïve Bayes, SMO, SVM, SVC, ANN, CHAID, and XGBoost to diagnose CAD. Unlike the abovementioned methods, this work proposes a new CAD diagnosis method based on eight different search techniques, principal component analysis (PCA), and AdaBoostM1. To the best of the author’s knowledge, there is no other work in the literature utilizing PCA and AdaBoostM1 techniques together for CAD diagnosis in this framework and detecting CAD based on age, hypertension, typical chest pain, t-wave inversion, and region with regional wall motion abnormality features. The major findings and contributions of this research study are as follows:

- Proposes a new method to diagnose CAD based on age, hypertension, typical chest pain, t-wave inversion, and region with regional wall motion abnormality features.
- Explores attribute spaces using eight different search methods, namely, evolutionary, best first, genetic, harmony, particle swarm optimization (PSO), greedy stepwise, rank, and multi-objective evolutionary search.
- Enhances the performance of CAD diagnosis by efficiently taking advantage of using PCA and AdaBoostM1 techniques together.
- The performance of the proposed method is tested in terms of several metrics and compared with basic classifiers and existing studies in the literature.
- Achieves the best classification performance ever reported on the Z-Alizadeh Sani dataset with so few features (five) with an accuracy rate of 91.80%.
- The experimental results demonstrate that the proposed method is promising to be utilized by medical specialists for diagnosing CAD.

The rest of the paper is ordered as follows. The proposed CAD diagnosis method and the dataset used are introduced in Section 2. Section 3 shows the experimental results, comparing the proposed method’s performance to the existing studies in the literature. Finally, conclusions are summarized in Section 4.

## 2. Materials and Methods

### 2.1. Dataset Description

In this work, the Z-Alizadeh Sani dataset that is freely available from the University of California—Irvine Machine Learning Repository [18] was used to evaluate the proposed method. The dataset contains 303 records, of which 87 of them are healthy persons and 216 of them are CAD patients. Fifty-five attributes can be classified into four groups: symptom and examination (14 attributes), demographic (17 attributes), electrocardiography (ECG) (7 attributes), and laboratory and echocardiography (echo) (17 attributes). The overview of the Z-Alizadeh Sani dataset, including attribute name, category, and range, is shown in Table 1.

### 2.2. The Proposed CAD Diagnosis Method

**Figure 3:**
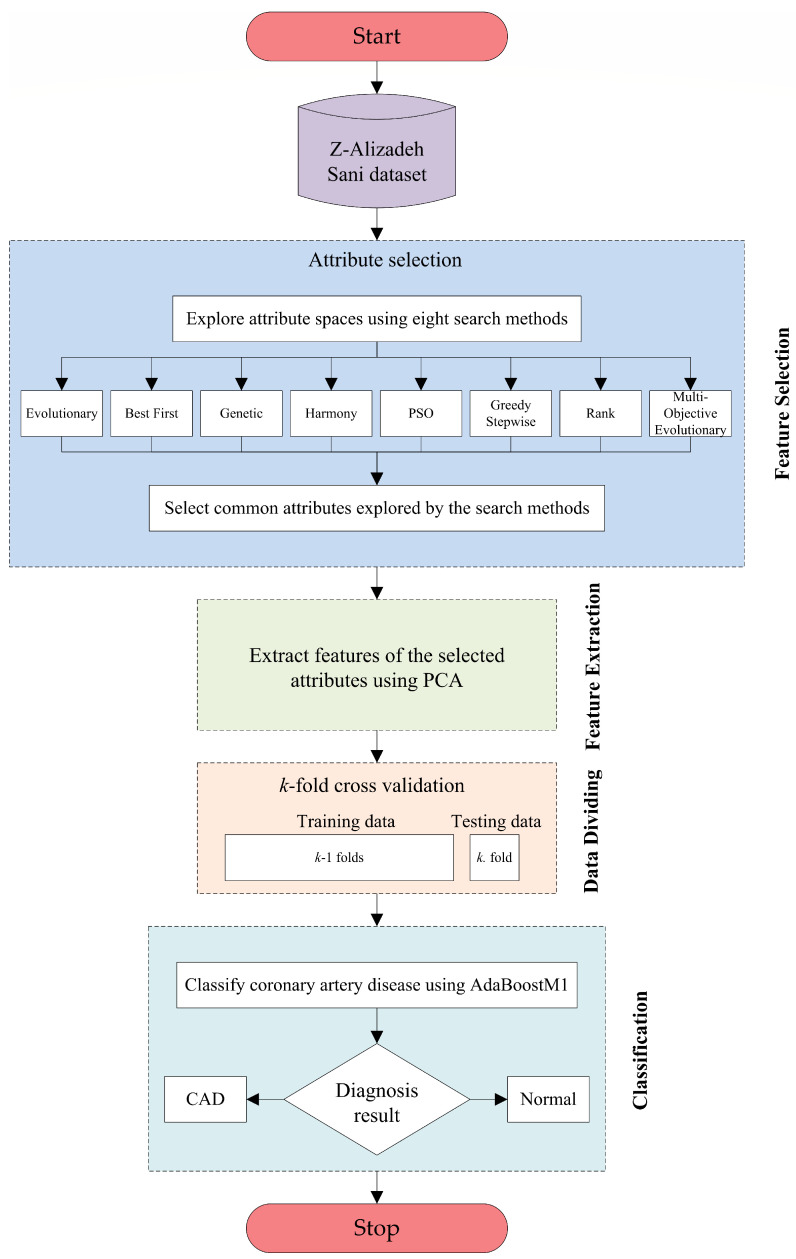
Flowchart of the proposed CAD diagnosis method.

This study presents a new CAD diagnosis method based on age, HTN, typical chest pain, t-wave inversion, and region-RWMA features. The proposed method comprises four basic steps, which are feature selection, feature extraction, data dividing, and classification. The flowchart of the proposed CAD diagnosis method is demonstrated in Figure 3. A correlation-based feature subset selection technique is utilized with evolutionary, best first, genetic, harmony, PSO, greedy stepwise, rank, and multi-objective evolutionary search methods in the feature selection step. Then, the PCA technique transforming the data into another space is used for feature extraction and size reduction on the data obtained after selecting common attributes. In the data-dividing step, the k-fold cross-validation technique is exploited to divide the whole dataset into k separate subsets, in which k-1 subsets are utilized for training and the remaining part is separated for testing. In the classification step, the AdaBoostM1 algorithm is performed for classifying coronary artery disease as healthy or patient. The techniques utilized to perform the proposed diagnostic method are described in the following subsections.

#### 2.2.1. Feature Selection

In the feature selection step of the proposed method, a correlation-based feature subset selection technique [37] was used with eight different search methods, namely, evolutionary [38], best first [39], genetic [40], harmony [41], PSO [42], greedy stepwise [43], rank [44], and multi-objective evolutionary search [45]. To evaluate the worth of a subset of attributes, the feature subset selection technique considers the estimative ability of every feature associated with the redundancy degree between them. The evolutionary search method utilizes an evolutionary algorithm (EA) to discover the attribute space. The best first search method uses a greedy hillclimbing algorithm enhanced with a backtracking ability for searching the space of a subset of attributes. The genetic search method carries out a search utilizing Goldberg’s genetic algorithm. The greedy stepwise search method applies a greedy backward/forward search, along with the space of a subset of attributes. To rank all the attributes, the rank search method utilizes a subset or attribute evaluator. Finally, the harmony, PS,O and multi-objective evolutionary search methods explore the attribute space using the harmony, PSO, and multi-objective evolutionary algorithms, respectively. Interested readers can kindly refer to [38,39,40,41,42,43,44,45] for further details about the search methods.

In the initial phase of the feature selection process outlined in the proposed method, eight distinct search methods are employed to explore and identify useful attributes. Table 2 presents the attributes selected through these search methods, along with the respective counts and attribute numbers. Subsequently, in the second stage, attributes common to all search methods are retained. As indicated in Table 2 (common attributes highlighted in bold), the selected attributes are numbered 1, 7, 25, 35, and 54, corresponding to features such as Age, HTN, Typical Chest Pain, T-Wave Inversion, and Region-RWMA.

#### 2.2.2. Feature Extraction

The data collected from a system often have dozens of related attributes. However, there may only be a few actual driving forces governing the behavior of a system, even though we have more attributes in the data measuring many system variables that provide redundant information [46]. It is usually possible to simplify problems containing redundancy by taking advantage of dimensionality reduction techniques. PCA is one of the most famous kinds of dimensionality reduction methods and has been widely used in various fields till now. It is intensely used for dimension reduction and feature extraction purposes as it decreases overfitting risk, reduces computational complexity, eliminates distracting noise, and so on [47].

PCA employs orthogonal transformations to condense multiple correlated variables into a reduced set of uncorrelated variables [47,48]. This technique establishes a new orthogonal-basis space where each axis represents a principal component, formed as a linear combination of the original data variables. By rigorously calculating these principal components, PCA ensures no redundancy of information within this new space [46]. Maximizing variance along each axis, PCA aligns the first axis with the highest variance of the data points, while the subsequent axes are orthogonal to the previous ones, sequentially maximizing the remaining variance [46]. Hence, in the transformed space, principal components are arranged in descending order of variance, with the first component explaining the most variance and subsequent components explaining progressively less [47,49].

The mathematical formulations required to compute the principal components are given hereafter. Let x(t) for t=1,2,⋯,n be an arbitrary dataset containing its corresponding instances and features with zero mean. Its covariance matrix R is computed as follows:

(1) $$\begin{array}{c} R=\frac{1}{n-1}\sum_{t=1}^{n}\left[x\left(t\right)x{\left(t\right)}^{T}\right] \end{array}$$

The next equation can be utilized to compute linear combinations of variables in the original data, i.e., the linear transformation from x(t) to y(t),

(2) $$\begin{array}{c} y\left(t\right)=M^{T}x\left(t\right) \end{array}$$

where *M* denotes an orthogonal matrix of the size n×n, and the *i*th column of this matrix, also of the sample covariance matrix *R*, is essentially equal to the *i*th eigenvector. At this point, the eigenvalue problem is initially set to be solved by the following equation:

(3) $$\begin{array}{c} \lambda_{l}q_{l}=Rq_{l} \end{array}$$

where $q_{l}$ represents the corresponding eigenvector, and $\lambda_{l}$ stands for an eigenvalue of the covariance matrix *R* (consider $\lambda_{1}>\lambda_{2}>\cdots >\lambda_{n}).$ Based on Equation (4), the principal component is computed by

(4) $$\begin{array}{c} y_{l}\left(t\right)=q_{l}^{T}x\left(t\right),\;l=1,\cdots ,n \end{array}$$

where $y_{l}\left(t\right)$ stands for the *i*th principle component. For additional information and further details, readers can refer to the references [47,48].

**Figure 4:**
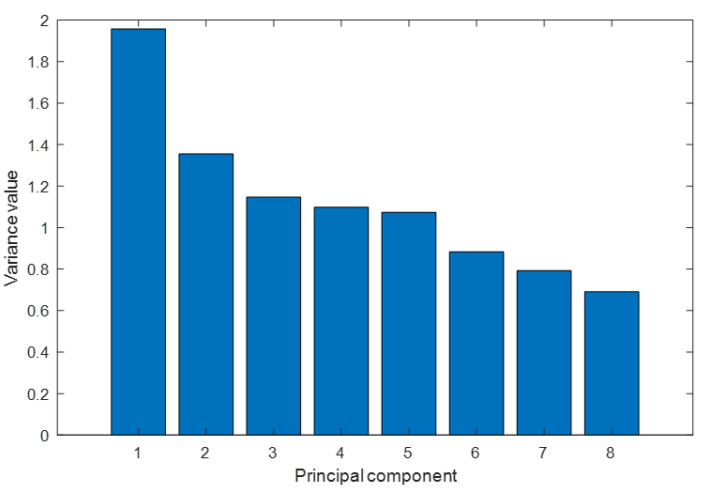
Variance values explained by each principal component.

For the Z-Alizadeh Sani dataset with selected attributes, Figure 4 illustrates the variance values explained by each principal component generated and depicts only the first eight components, which account for around 95% of the total variance.

#### 2.2.3. Data Dividing

A methodology known as k-fold cross-validation can be used to reduce the bias related to a random sampling of the holdout and training data samples when comparing the predicted accuracy of two or more methods. The entire data set is randomly separated into k mutually exclusive subsets of approximately similar size in k-fold cross-validation, also known as rotation estimation. The classification technique is trained and tested k times. k-1 of mutually exclusive subsets are utilized for training, while the remaining one is reserved for testing. With averaging the k individual accuracy measures, the prediction of a technique’s overall accuracy is computed as

(5) $$\begin{array}{c} CV\;accuracy=\frac{1}{k}\sum_{i=1}^{k}A_{i} \end{array}$$

where *A* represents the accuracy measure of each fold such as specificity, sensitivity, and hit rate, and *k* denotes the number of used folds [50,51].

**Figure 5:**
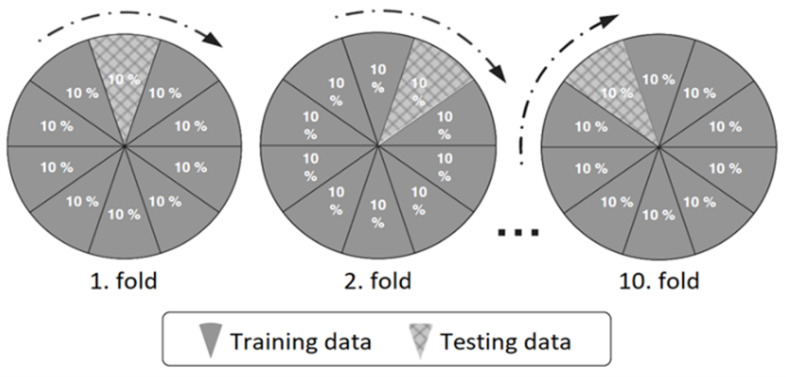
Visualization of 10-fold cross-validation.

Since it is the most widespread practice for *k* to have a value of 10, the k-fold cross-validation is also known as 10-fold cross-validation. Empirical studies have shown that the optimal number of folds seems to be 10 [50,51]. For this reason, in this study, 10-fold cross-validation was utilized for evaluating the proposed diagnosis method. Figure 5 shows a visualization of k-fold cross-validation with k=10 [50,51].

#### 2.2.4. Classification

In the classification step of the proposed method, the AdaBoostM1 algorithm [52] is utilized to classify coronary artery disease as patient or normal. The following is a description of the AdaBoostM1 algorithm. $T_{n}=\left\{\left(X_{1},Y_{1}\right),\left(X_{2},Y_{2}\right),\cdots ,\left(X_{n},Y_{n}\right)\right\}$ is a training set with *Y* values in 1,2,⋯,k. Each observation $X_{i}$ is given a weight $w_{b}\left(i\right)$, which is originally set to 1/n. After each step, this value is updated. The classifier’s error is denoted by $\epsilon_{b}$ and is calculated as follows:

(6) $$\begin{array}{c} \epsilon_{b}=\sum_{i=1}^{n}\left[w_{b}\left(i\right)\xi_{b}\left(i\right)\right] \end{array}$$

where

(7) $$\begin{array}{c} \xi_{b}=\left\{\begin{array}{cc} 0, & C_{b}\left(x_{i}\right)=y_{i}\\ 1, & C_{b}\left(x_{i}\right)\neq y_{i} \end{array}\right. \end{array}$$

The constant $\alpha_{b}$ is calculated from the classifier’s error in the *b*th iteration, and this value is utilized for the weight update. Particularly, $\alpha_{b}=1/2ln\left(\left(1-\epsilon_{b}\right)/\epsilon_{b}\right)$, and for the b+1th iteration, the new weight is

(8) $$\begin{array}{c} w_{b+1}\left(i\right)=w_{b}\left(i\right)\exp\left\{\alpha_{b}\xi_{b}\left(i\right)\right\} \end{array}$$

The obtained weights are then normalized to the sum of one. As a result, the weight of incorrectly categorized observations increases while the weight of correctly classified observations reduces, driving the single classifier produced in the next iteration to focus on the most difficult examples. Furthermore, while the single classifier’s error is low, differences in weight updates are bigger, since when the classifier gets a high accuracy, the few mistakes become more important. Thus, the alpha constant can be thought of as a learning rate computed as a function of each iteration’s mistake. Additionally, this constant is employed in the final decision rule, which gives more weight to the individual classifiers with the lowest error. This process is repeated in each step for b=1,2,3,⋯,B. Finally, the ensemble classifier calculates the weighted sum of each class’s votes. As a result, the class with the highest weighted vote receives the assignment. In particular [52,53],

(9) $$\begin{array}{cc} C(x) & =\arg_{y_{j}}\max\sum_{b=1}^{B}\left[\alpha_{b}\delta \left(C_{b}\left(x\right),y_{j}\right)\right] \end{array}$$

(10) $$\begin{array}{cc} \; & =\arg_{y_{j}}\max\sum_{b:C_{b}\left(x\right)=y_{j}}\alpha_{b} \end{array}$$

## 3. Experimental Results and Discussions

### 3.1. Performance Metrics

**Figure 6:**
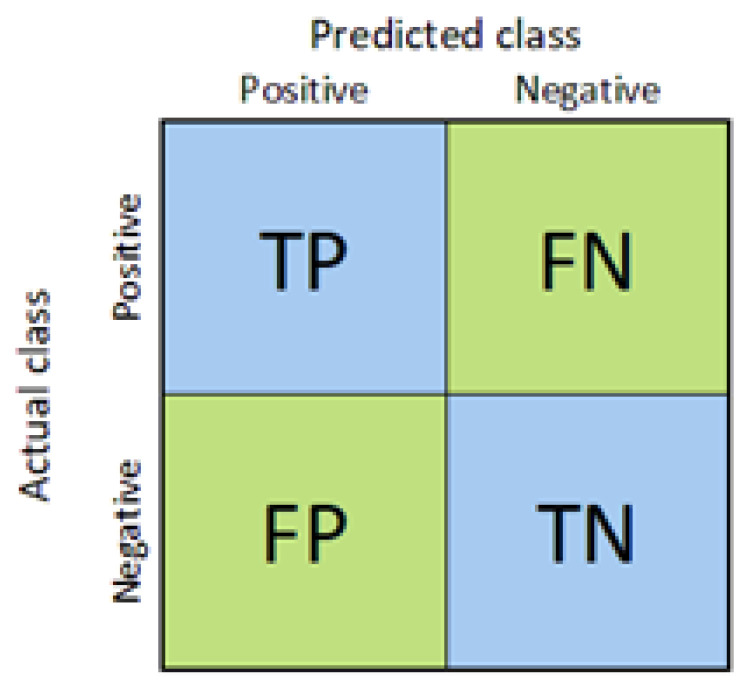
Confusion Matrix.

In this study, to measure the proposed CAD diagnosis method’s effectiveness, various basic metrics, which are accuracy, precision, recall, $F_{1}$, AUC, and MCC, are employed, and these metrics are computed as follows [54,55,56,57]:

(11) $$\begin{array}{cc} Accuracy & =\frac{TP+TN}{TP+TN+FP+FN} \end{array}$$

(12) $$\begin{array}{cc} Precision & =\frac{TP}{TP+FP} \end{array}$$

(13) $$\begin{array}{cc} Recall & =\frac{TP}{TP+FN} \end{array}$$

(14) $$\begin{array}{cc} F_{1} & =\frac{2\cdot Precision\cdot Recall}{Precision+Recall} \end{array}$$

(15) $$\begin{array}{cc} AUC & =\frac{1}{2}\left(\frac{TP}{TP+FN}+\frac{TN}{TN+FP}\right) \end{array}$$

(16) $$\begin{array}{cc} MCC & =\frac{TP\cdot TN-FP\cdot FN}{\sqrt{\left(TP+FP)\cdot (TP+FN)\cdot (TN+FP)\cdot (TN+FN\right)}} \end{array}$$

where the types of possible outcomes are TP (true positives—correctly labeled as positive tuples), TN (true negatives—correctly labeled as negative tuples), FP (false positives—negative tuples incorrectly labeled as positive), and FN (false negatives—positive tuples mislabeled as negative) in binary estimation [54,58]. The confusion matrix, a summary of the possible outcomes, is demonstrated in Figure 6.

### 3.2. Experiments on the Feature Extraction

The initial experiment focused on the feature extraction method employed in the proposed approach. Two distinct techniques were utilized: exclusive feature selection and PCA. The performance metrics results are presented in Table 3, utilizing various data-division methodologies, including an 80% training–20% test split, and 5-fold and 10-fold cross-validation. Notably, employing feature selection alone yielded a classification accuracy of 90.164% in the 80% training–20% test data division. Furthermore, precision, recall, F-measure, and AUC metrics exceeded 0.9, with a Matthews correlation coefficient (MCC) rate of 0.755. Subsequently, integrating PCA with the selected features led to improved accuracy, precision, recall, F-measure, and MCC metrics, resulting in an accuracy rate of 91.803%, as demonstrated in Table 3.

In the data-dividing methodology of 5-fold cross-validation, an accuracy of 86.799% was attained, and results in the range of 0.893 to 0.926 were obtained for precision, recall, F-measure, and AUC. When the PCA technique was utilized for feature extraction, some rise was observed in accuracy, precision, recall, F-measure, and MCC metrics. Finally, in 10-fold cross-validation, the use of the PCA technique increased by approximately three percent in the accuracy metric. Moreover, the results of precision, recall, F-measure and M,CC metrics rose. Considering all the results, the best classification accuracy rate of 91.803%, precision rate of 0.933, recall rate of 0.955, F-measure rate of 0.944, and MCC rate of 0.793 were achieved in 80% training–20% test splitting methodology when PCA was used. Additionally, the best AUC rate of 0.929 was achieved with the feature extraction technique of feature selection only.

**Figure 7:**
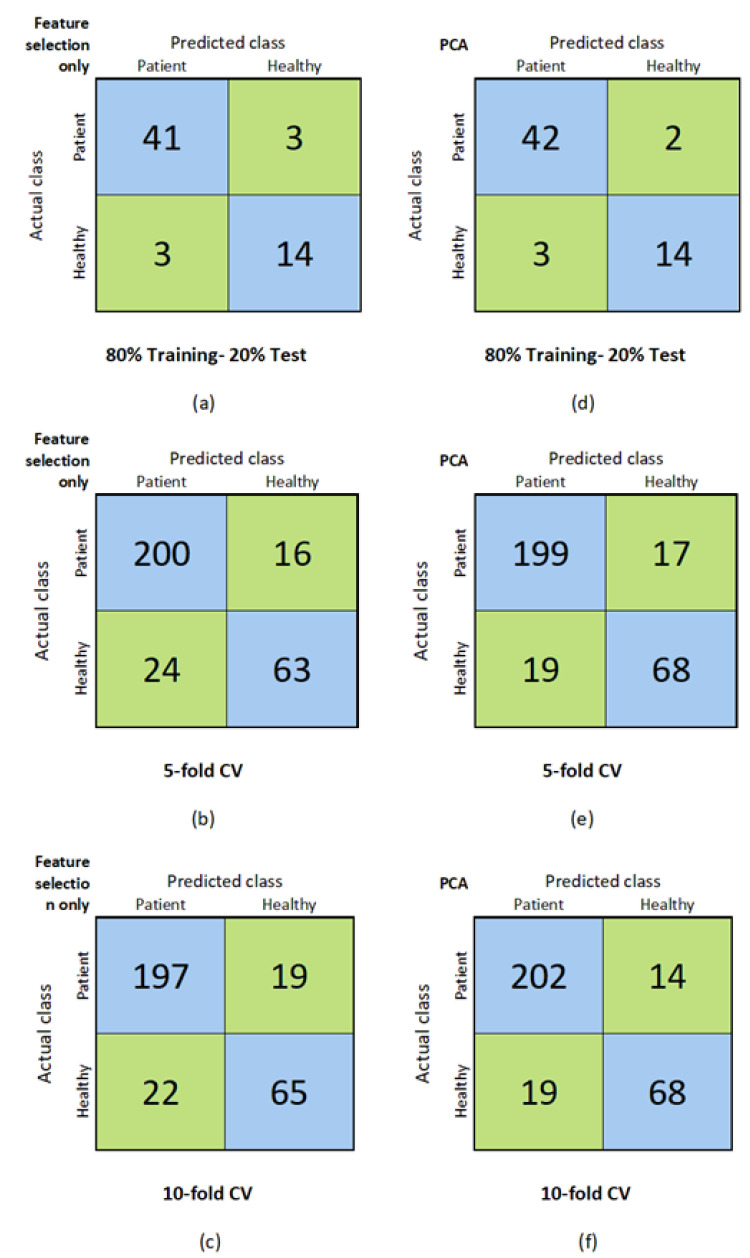
Confusion matrices for each feature extraction technique and data-dividing methodology. Figures in (**a**–**c**) refer to “Feature-selection-only” results for 80%-Training-20%Test, 5-fold-CV and 10-fold-CV, respectively. Similarly, (**d**–**f**) refer to the same results respectively but this time for the “PCA” method.

In addition, the confusion matrices for each feature extraction technique and data-dividing methodologies are given in Figure 7. The confusion matrices acquired for the feature selection only are depicted in Figure 7a–c while Figure 7d–f demonstrate the confusion matrices attained for the PCA technique with the data-dividing methodologies of 80% training–20% test and 5-fold and 10-fold cross-validation, respectively.

### 3.3. Comparison with Traditional Methods

This subsection compares the classification results of the proposed method with basic classifiers. On the Z-Alizadeh Sani dataset, several basic techniques were tested in the 10-fold cross-validation. The aforementioned two extraction techniques of feature selection only and PCA were utilized with each basic classifier, and their performance results are shown with regard to the previously mentioned six metrics in Table 4. Along with the proposed approach, this table contains the results of eleven basic classifiers such as Naïve Bayes [59], k-NN (k = 5) [60], C4.5 decision tree [61], locally weighted learning (LWL) [62], K* [63], logistic model trees (LMT) [64], SVM [65], random forests [66], logistic regression [67], Hoeffding tree [68], and deep learning 4J [69]. As can be seen from the table, using PCA to extract the features increased the classification accuracy performance of k-NN, C4.5 decision tree, LWL, SVM, deep learning 4J and the proposed method. LMT and logistic regression with an accuracy of 88.449% are the best classifiers for feature selection only, whereas the proposed method with PCA achieves the best accuracy rate of 89.109%, a recall rate of 0.935, and an F-measure rate of 0.924, surpassing the other techniques.

### 3.4. Comparison with Existing Methods in the Literature

In this subsection, the proposed method was compared with the existing studies in the literature using the same dataset, the Z-Alizadeh Sani dataset. The performance comparison of the proposed method with the existing works is presented in Table 5, containing researcher names, years, used method, number of selected features, and accuracy metrics. Between 2012 and 2016, Alizadehsani et al. [19,20,21,22,23,24,25] used different numbers of features, such as 16, 20, 24, and 34, and achieved the best accuracy of 94.08% utilizing information gain and SMO. In 2017, Qin et al. [26] applied their CAD detection approach based on EA-MFS and SVM with 34 features and procured an accuracy rate of 93.70%. In the same year, Arabasadi et al. [27] proposed a genetic algorithm and MLP-ANN-based CAD prediction method selecting 22 features, while Babič et al. [28] performed various classifiers such as decision trees, Naïve Bayes, SVM, and ANN and used 27 features to feed these classifiers.

In 2018, Kılıç and Kaya Keleş [29] selected 16 features using the ABC algorithm and then classified CAD utilizing the SMO technique. As a result of their study, an accuracy rate of 89.44% was obtained. In 2019, MML-IBLMM and Var-IBLMM methods introduced by Hu et al. [30] were applied to the Z-Alizadeh Sani dataset and attained an accuracy rate of 81.84%. In the same year, Abdar et al. [31] proposed the N2Genetic-nuSVM approach, selected 29 features, and acquired an accuracy rate of 93.08%. In another work performed by Abdar et al. [32], a CAD diagnosis approach called NE-nu-SVC was presented. In this approach, 16 features were selected and an accuracy of 94.66% was achieved.

In 2020, Joloudari et al. [33] tested the classification performance of various classifiers, selected 40 features, and obtained the best accuracy rate of 91.47% with random trees. In the same year, a hybrid feature selection method called 2HFS was introduced by Nasarian et al. [34], and 38 features were selected using this method. In the sequel, SMOTE and XGBoost techniques were used together and an accuracy rate of 92.58% was reported. In another study presented by Ashish et al. [35], a random forests-, SVM-, and XGBoost-based CAD detection approach was implemented and an accuracy rate of 93.86% was achieved with 10 features. In a recent work [36], 25 features were used, and an accuracy rate of 91.78% with the MLP classifier was obtained.

Unlike these studies, the proposed method in this work utilizes five features, namely, age, hypertension, typical chest pain, t-wave inversion, and region with regional wall motion abnormality. In the dataset with these features, PCA and AdaBoostM1 techniques were used for feature extraction and classification, respectively. The best accuracy of 91.80% was achieved when using these few features on the Z-Alizadeh Sani dataset.

### 3.5. Limitations

This work can be considered a retrospective study because it uses a dataset based on past patient records. Researchers conduct this type of study by examining the existing records, historical data, or previous occurrences in order to determine outcomes, relationships, or correlations between variables. In contrast to prospective studies, which follow participants ahead of time, retrospective studies begin with the desired outcome or endpoint and go backwards to investigate the reasons or events that led to it. For example, in a prospective study introduced by Locuratolo et al. [70], patients were evaluated clinically and in the laboratory after 30 days, 3 months, 6 months, and 1 year following the index incident. Various endpoints related to acute coronary syndrome were evaluated. At the end of the study, the persistence of treatments and the percentage of patients who achieved therapeutic goals were evaluated.

Retrospective studies have some limitations, such as data quality, limited scope, bias, temporal ambiguity, confounding variables, validity of exposure measurement, and causality inference. In spite of these limitations, retrospective studies remain useful in epidemiological research, particularly when prospective investigations are unfeasible or unethical. The method proposed in this study can help medical doctors diagnose CAD early by using a small number of features.

## 4. Conclusions

This paper introduces an effective approach for diagnosing coronary artery disease (CAD) by leveraging age, hypertension, typical chest pain, T-wave inversion, and regional wall motion abnormality features. The method proposed utilizes eight distinct search techniques, including evolutionary, best first, genetic, harmony, PSO, greedy stepwise, rank, and multi-objective evolutionary search, to perform feature selection on the Z-Alizadeh Sani dataset. Principal component analysis (PCA) and the AdaBoostM1 algorithm are employed for feature extraction and CAD classification, respectively. Through extensive experiments and analyses using various performance metrics, the proposed method achieves the highest prediction performance to date with only five attributes. Notably, it achieves impressive accuracy, precision, recall, F-measure, AUC, and MCC rates of 91.8%, 93.3%, 95.5%, 94.4%, 89.5%, and 79.3%, respectively. These results demonstrate the efficiency of the proposed approach and its potential to serve as a cost-effective tool to aid medical practitioners in CAD diagnosis. 

## Figures and Tables

**Table 1:** The Overview of Z-Alizadeh Sani Dataset.

#	Attribute Name	Attribute Category	Attribute Range
1	Age	Demographic	30–86
2	Weight	Demographic	48–120
3	Length	Demographic	140–188
4	Sex	Demographic	Female, Male
5	Body Mass Index (BMI, Kg/m^2^)	Demographic	18.115–40.901
6	Diabetes Mellitus (DM)	Demographic	Yes, No
7	Hypertension (HTN)	Demographic	Yes, No
8	Current Smoker	Demographic	Yes, No
9	Ex-Smoker	Demographic	Yes, No
10	Family History (FH)	Demographic	Yes, No
11	Obesity (BMI > 25)	Demographic	Yes, No
12	Chronic Renal Failure (CRF)	Demographic	Yes, No
13	Cerebrovascular Accident (CVA)	Demographic	Yes, No
14	Airway Disease	Demographic	Yes, No
15	Thyroid Disease	Demographic	Yes, No
16	Congestive Heart Failure (CHF)	Demographic	Yes, No
17	Dyslipidemia (DLP)	Demographic	Yes, No
18	Blood Pressure (BP, mmHg)	Symptom and examination	90–190
19	Pulse Rate (PR, ppm)	Symptom and examination	50–110
20	Edema	Symptom and examination	Yes, No
21	Weak Peripheral Pulse	Symptom and examination	Yes, No
22	Lung Rales	Symptom and examination	Yes, No
23	Systolic Murmur	Symptom and examination	Yes, No
24	Diastolic Murmur	Symptom and examination	Yes, No
25	Typical Chest Pain	Symptom and examination	Yes, No
26	Dyspnea	Symptom and examination	Yes, No
27	Function Class	Symptom and examination	0, 1, 2, 3
28	Atypical	Symptom and examination	Yes, No
29	Nonanginal Chest Pain	Symptom and examination	Yes, No
30	Exertional Chest Pain	Symptom and examination	Yes, No
31	Low Threshold Angina (Low TH Ang)	Symptom and examination	Yes, No
32	Q-Wave	ECG	Yes, No
33	ST Elevation	ECG	Yes, No
34	ST Depression	ECG	Yes, No
35	T-Wave Inversion	ECG	Yes, No
36	Left Ventricular Hypertrophy (LVH)	ECG	Yes, No
37	Poor R-Wave Progression	ECG	Yes, No
38	Bundle Branch Block (BBB)	ECG	Left, Right, Normal
39	Fasting Blood Sugar (FBS, mg/dL)	Laboratory and echo	62–400
40	Creatine (Cr, mg/dL)	Laboratory and echo	0.5–2.2
41	Triglyceride (TG, mg/dL)	Laboratory and echo	37–1050
42	Low Density Lipoprotein (LDL, mg/dl)	Laboratory and echo	18–232
43	High Density Lipoprotein (HDL, mg/dL)	Laboratory and echo	15.9–111
44	Blood Urea Nitrogen (BUN, mg/dL)	Laboratory and echo	6–52
45	Erythrocyte Sedimentation Rate (ESR, mm/h)	Laboratory and echo	1–90
46	Hemoglobin (HB, g/dL)	Laboratory and echo	8.9–17.6
47	Potassium (K, mEq/lit)	Laboratory and echo	3–6.6
48	Sodium (Na, mEq/lit)	Laboratory and echo	128–156
49	White Blood Cell (WBC, cells/mL)	Laboratory and echo	3700–18,000
50	Lymphocyte (%)	Laboratory and echo	7–60
51	Neutrophil (%)	Laboratory and echo	32–89
52	Platelet (PLT, 1000/mL)	Laboratory and echo	25–742
53	Ejection Fraction (%)	Laboratory and echo	15–60
54	Region-RWMA	Laboratory and echo	0, 1, 2, 3, 4
55	Valvular Heart Disease (VHD)	Laboratory and echo	Mild, Severe, Moderate, Normal

**Table 2:** The Attributes Chosen Using Search Methods. Bold attribute numbers refer to the common features for all search methods.

Search Method	Number of Chosen Attributes	Attribute No.
Evolutionary	17	**1**, **7**, 9, 14, 15, 18, 24, **25**, 28, 29, 31, **35**, 39, 41, 45, 47, **54**
Best first	12	**1**, 6, **7**, 18, **25**, 28, 29, **35**, 45, 47, 53, **54**
Genetic	15	**1**, 4, 6, **7**, 12, 18, **25**, 28, 29, 32, 34, **35**, 47, 53, **54**
Harmony	17	**1**,**7**, 12, 13, 14, 15, 17, **25**, 27, 28, 29, **35**, 37, 45, 47, 53, **54**
PSO	14	**1**, 6, **7**, 18, **25**, 28, 29, 32, 34, **35**, 45, 47, 53, **54**
Greedy stepwise	10	**1**, 6, **7**, 14, **25**,**35**, 45, 47, 53, **54**
Rank	13	**1**, 6, **7**, 14, **25**, 28, 29, 32, 33, **35**, 45, 53, **54**
Multi-objective evolutionary	10	**1**, 6, **7**, 14, **25**, **35**, 45, 47, 53, **54**

**Table 3:** Performance Metric Results of Various Feature Extraction Techniques with AdaBoostM1.

Feature Extraction Technique		80/20% Train/Test Split	5-Fold CV	10-Fold CV
Feature selection only	%Acc.	90.164	86.799	86.469
	Precision	0.932	0.893	0.900
	Recall	0.932	0.926	0.912
	F1	0.932	0.909	0.906
	AUC	0.929	0.909	0.907
	MCC	0.755	0.670	0.666
PCA	%Acc.	91.803	88.119	89.109
	Precision	0.933	0.913	0.914
	Recall	0.955	0.921	0.935
	F1	0.944	0.917	0.924
	AUC	0.895	0.888	0.879
	MCC	0.793	0.708	0.730

**Table 4:** Classification Performance Results Of Basic Classifiers. Bold-faced results refer to the best performing results for each metric.

Feature Extraction Technique		Naïve Bayes [59]	k-NN [60]	C4.5 DT [61]	LWL [62]	K* [63]	LMT [64]	SVM [65]	RF [66]	Log Reg [67]	Hoeff. Tree [68]	DL 4J [69]	Ours
Feature selection only	%Acc.	88.120	85.480	85.150	87.130	83.500	88.450	87.790	81.520	88.450	87.790	85.480	86.470
	Preci.	0.905	0.910	0.890	0.900	0.877	0.910	0.916	0.864	0.910	0.905	0.939	0.900
	Recall	0.931	0.884	0.903	0.921	0.894	0.931	0.912	0.880	0.931	0.926	0.852	0.912
	F1	0.918	0.897	0.897	0.911	0.885	0.920	0.914	0.872	0.920	0.915	0.893	0.906
	AUC	0.923	0.894	0.830	0.907	0.901	0.919	0.853	0.881	0.922	0.923	0.922	0.907
	MCC	0.705	0.653	0.634	0.681	0.592	0.714	0.703	0.543	0.714	0.697	0.676	0.666
PCA	%Acc.	80.530	86.470	87.790	87.790	81.850	86.800	88.120	81.190	88.450	80.200	86.800	**89.110**
	Preci.	0.920	0.900	0.912	0.905	0.871	0.889	0.917	0.863	0.910	0.919	**0.944**	0.914
	Recall	0.796	0.912	0.917	0.926	0.875	0.931	0.917	0.875	0.931	0.792	0.866	**0.935**
	F1	0.854	0.906	0.915	0.915	0.873	0.910	0.917	0.869	0.920	0.851	0.903	**0.924**
	AUC	0.892	0.878	0.846	0.697	0.858	0.918	0.855	0.874	**0.922**	0.892	0.921	0.879
	MCC	0.581	0.666	0.701	**0.885**	0.555	0.668	0.710	0.536	0.714	0.575	0.703	0.730

**Table 5:** Performance Comparison of The Proposed Method with The Existing Studies using the Z-Alizadeh Sani Dataset.

Paper	Year	Method	# of Features	Accuracy (%)
[22]	2012	SMO	16	82.16
[25]	2012	Naïve Bayes–SMO	16	88.52
[21]	2012	SMO	34	92.09
[24]	2012	SMO 1-1	34	92.74
[19]	2013	Information gain + SMO	34	94.08
[20]	2013	Bagging	20	79.54 (LAD)
		+		61.46 (LCX)
		C4.5		68.96 (RCA)
[23]	2016	Average and combined	24	86.14 (LAD)
		information gain		83.17 (LCX)
		+ SVM		83.50 (RCA)
[26]	2017	EA-MFS + SVM	34	93.7
[27]	2017	GA + MLP-ANN	22	93.85
[28]	2017	SVM	27	86.67
[29]	2018	ABC + SMO	16	89.44
[30]	2019	MML-IBLMM and Var-IBLMM	55	81.84
[31]	2019	N2Genetic-nuSVM	29	93.08
[32]	2019	NE-nu-SVC	16	94.66
[33]	2020	Random trees	40	91.47
[34]	2020	2HFS + SMOTE + XGBoost	38	92.58
[35]	2021	Random forests + SVM + XGBoost	10	93.86
[36]	2023	MLP	25	91.78
**Proposed**		**PCA + AdaBoostM1**	**5**	**91.8**

## Data Availability

This paper utilised the Z-Alizadeh Sani dataset that is freely available from the University of California—Irvine Machine Learning Repository [18].
